# Assessment of The Effect of Stress, Sociodemographic Variables and Work-Related Factors on Rationing of Nursing Care

**DOI:** 10.3390/ijerph20032414

**Published:** 2023-01-29

**Authors:** Daria Schneider-Matyka, Natalia Świątoniowska-Lonc, Jacek Polański, Małgorzata Szkup, Elżbieta Grochans, Beata Jankowska-Polańska

**Affiliations:** 1 Department of Nursing, Pomeranian Medical University in Szczecin, Żołnierska 48, 71-210 Szczecin, Poland; 2 Center for Research and Innovation, 4th Military Hospital, 5 Weigla Street, 50-981 Wrocław, Poland; 3 Department of Internal Medicine, Occupational Diseases, Hypertension and Clinical Oncology, Wroclaw Medical University, 213 Borowska Street, 50-556 Wrocław, Poland

**Keywords:** rationing of nursing care, stress, job satisfaction

## Abstract

(1) Rationing of nursing care is withholding, limiting or not fulfilling the necessary nursing activities for patients. It may have a negative effect on patient safety and the quality of care. The aim of the present paper is the assessment of the effect of stress on the rationing of nursing care. (2) The current research has a cross-sectional, observational design. The study sample comprised 567 nurses. The following questionnaires were used in the study PIRNCA and PSS-10. (3) It was demonstrated that nurses with a high level of perceived stress rationed nursing care to a greater extent and showed lower assessment of nursing care and lower job satisfaction in comparison with nurses with average and low levels of perceived stress. (4) 1. Stress in nurses has a negative effect on rationing of nursing care and job satisfaction. It is recommended that actions aimed at developing effective stress coping skills be implemented as early as at the stage of training to become a nurse. 2. Factors such as marital status, sex, form of employment, place of employment and the level of professional burnout syndrome may have an influence on the level of experienced stress. In turn, the level of experienced stress, marital status, education, place of work as well as the place of residence may have an effect on rationing of nursing care and, consequently, affect the quality of care.

## 1. Introduction

In general, rationing is defined as controlled and cautious allocation of limited resources when the demand exceeds the supply [1]. The phenomenon of rationing of nursing care was first described in 2006 by American nurse Beatrice Kalisch [2]; in Poland the issue was first presented by Uchmanowicz et al. [3]. Rationing of nursing care is understood as withholding, limiting or omitting the necessary nursing activities towards patients owing to lack of nursing resources (personnel, skills, time) [4]. In light of the wide availability of health care and limited resources to provide it, rationing of nursing care has become difficult to avoid [5,6]. Numerous factors affect rationing of nursing care, not only those relating to a patient or a nurse but also work environment and organisational resources. It is believed that rationing of nursing care stems from, among other factors, shortage and rotation of medical staff, unsatisfactory salary, heavy workload (unexpected increase in the number of patients and intensity of care, greater number of patient admissions and discharges, inadequate support from the auxiliary personnel and other emergencies affecting the organisation of work), conflicts within the therapeutic teams, inadequate working conditions, lack of support from the management staff and lack of possibility of participating in the clinical decision-making process [7,8]. Rationing of nursing care may have a negative effect on patient safety and the quality of care [9,10]. Consequently, it may lead to the occurrence of adverse events, lowering the quality of provided care, risks to patient safety such as increased mortality, errors in the medication administration process, urinary tract infections, patient falls, pressure sores and patient readmission. With respect to the safety of the patient, rationing of nursing care is just as harmful as medical errors. The nursing staff participates in most diagnostic and therapeutic procedures and, consequently, has a major effect on the quality of care [10,11]. Nurses experience a feeling of guilt if they are unable to provide the required care to all patients and this, ultimately, leads to stress, professional burnout syndrome and the need to rotate [12]. In turn, rotation of nursing staff may be an additional factor in the higher level of rationing of nursing care [13]. 

Nursing is considered a profession exposed to a high risk of stress due to, among other factors, fast pace of work and the constant need to cope with emergencies. The issue of professional burnout syndrome in nurses is frequently discussed as it is directly related to experiencing stress. Nurses are exposed to psychological stress and are therefore more susceptible to fatigue than other health care workers [14]. The studies conducted on 2355 nurses in Italy showed that 65.4% of the respondents suffered from insomnia, which may be linked to shift work, being under much pressure, workload and high professional risk [15]. Nowadays, stress experienced at work accounts for 50–80% of psychosomatic diseases or conditions related to stress among employees [14].

Work-related stress is defined as a harmful psychobiological reaction to a situation in which work requirements are not in line with the possibilities, resources or the needs of an employee. This may be related to work itself (workload, poor decision-making skills) but also with work organisation and environment (poor communication, interpersonal conflicts) as well as difficulties in reconciling family life with work. Individual personality traits and different styles of coping with stress explain significant variability in the perception and response to work requirements and environment. Work-related stress is associated with numerous negative effects on physical and mental health and is becoming an increasing problem among healthcare workers [16,17,18]. In the nursing profession, work-related stress frequently stems from personnel shortage. As a result, nurses are charged with too many responsibilities and are incapable of providing equal care to all patients. Work overload leads to haste and lack of verbal contact with a patient and may result in rationing of nursing care. Often, it is accompanied by lack of support from superiors, low salary and lack of career advancement opportunities [19].

Psychosocial factors cause stress in nurses which, when combined with fatigue, can affect the level of job satisfaction and quality of care, thus resulting in rationing of nursing care. The relationship between the level of stress, job satisfaction and rationing of care may be different for individual nurses and show different intensity. The studies conducted among students and health care workers indicate the relationship between stress and reduced functioning. It has been demonstrated that high levels of support translate into improved results [20]. Nursing students experience stress as early as at the stage of professional clinical training [21,22]. The most commonly reported stressors include the feeling of having inadequate knowledge or skills, fear of making errors resulting in patient harm, heavy workload [22,23,24], lack of support or feedback from supervisors [22,25,26] as well as ineffective work organisation during classes [27]. Identifying the sources of stress experienced by nursing students during clinical training would allow support in coping with stress to be provided [28]. Systematic evaluation and implementing corrective action can achieve a comprehensive, complete and unrestricted nursing care. 

The aim of the present paper is the assessment of the effect of stress on rationing of nursing care.

## 2. Materials and Methods

The current research has a cross-sectional, observational design. The sample included 567 nurses working in Lower Silesia, Mazovia and West Pomerania provinces in Poland. Data for this study were collected from September 2020 to December 2021. The study was conducted either directly by distributing questionnaires or electronically by granting access to an electronic questionnaire via the link. Trained persons responsible for collecting the questionnaires invited nurses from selected wards or facilities to participate in the study, having obtained the approval from senior management. Once the consent to participate in the study was obtained, the instructions, information on the study and the link to an electronic version of the questionnaire were made available via the intranet. Nurses’ supervisors were asked to regularly remind the nurses of the questionnaire. In facilities without the intranet, it was possible to fill in the questionnaire in paper version or electronically via the link sent to a private email. The crucial element of the study was completing the questionnaires personally. In total, approximately 800 nurses working in facilities selected for the study were invited to participate in the study. The selection of facilities was random and determined by the possibility of cooperation with the senior management, which allowed expedite consent to the study without the long waiting period. Unfortunately, not all of the invited nurses participated in the study. In total, 616 questionnaires were received, although some of them were incomplete and were excluded from the study (49 questionnaires). 

The inclusion criterion was work experience in the position of a nurse of at least 6 months. The exclusion criteria were lack of consent to participate in the study and work experience shorter than 6 months. The nurses who were receiving psychiatric help or undergoing pharmacological treatment for depression were also excluded from the study. Participation was voluntary and anonymous. 

Data were collected using a proprietary survey questionnaire consisting of 11 questions constituting the metrics. The following standardized questionnaires were used in the study:

The Perceived Implicit Rationing of Nursing Care (PIRNCA) [29] for the assessment of unfinished nursing activities (direct or delegated) during the last seven working shifts [30]. The questionnaire comprises 31 questions, allowing the assessment of 6 main areas of nursing activities: nursing care, implementing the prescribed treatment plan, emotional support and education, supervision/alertness, coordination of care and planning, documenting the undertaken activities as well as two questions, analysed separately, concerning the quality of nursing care and job satisfaction. The respondents declare how often in the last 7 working days they were unable to perform the activities listed in the questionnaire. The response to each question is provided using one of the answers: never = 0, rarely =1, sometimes =2, often =3. The total score is the average score of the answers provided by the respondents (the questions marked as “not applicable” are excluded). The total score is 0–3 and can be interpreted as follows: higher score means more frequent implicit rationing of care. The higher the score, the higher the level of rationing of care. The answers to the questions concerning the nurses’ assessment of patient care and overall job satisfaction are given using 0–10 scale, and higher scores indicate better quality of care and higher level of job satisfaction. The Polish adaptation of the questionnaire showed very high psychometric properties (Cronbach’s alpha for the entire questionnaire 0.957) [19].

For the purpose of assessing the level of stress related to life events over the past month, the Perceived Stress Scale (PSS-10) was used [31]. The test comprises 10 questions concerning various subjective appraisals of personal problems and events, stress-coping behaviours and methods. The internal consistency of the Polish adaptation of the questionnaire amounted to Cronbach’s alpha = 0.86. The respondents answer the questions by providing a number (0—never, 1—almost never, 2—sometimes, 3—fairly often, 4—very often). The total score is a sum of all answers with a theoretical distribution of 0 to 40. The higher the score, the higher the level of perceived stress. The overall index, once transformed into standardised units, is interpreted according to the respective properties of the sten scale. The results from 1 to 4 stens are considered low, whereas 7–10 is considered high. The results 5–6 stens are considered average [32].

### Statistical Analysis

Statistical analysis was conducted using Statistica 13.1 software (TIBCO Inc., Palo Alto, CA, USA). For measurable variables, the following were calculated: arithmetic mean, median, standard deviation, variability range (extreme values). For qualitative variables, the frequency of occurrence was calculated (percent). All quantitative variables under analysis were analysed using the Shapiro–Wilk test to determine the distribution type. To determine the differences between the groups, a non-parametric Kruskal–Wallis ANOVA test was used. Spearman’s rank correlation between the selected variables was conducted and analysed. The significance level for all comparisons was set at α= 0.05.

## 3. Results

The study group comprised 567 nurses (the average age 42.2 years; SD = 10.8), employed in primary health care facilities and in hospital wards of various profiles. The largest group of respondents were women—75.3%, city residents—83.1% and being in a relationship –78.4%. The statistical analysis showed that the largest percentage of the respondents had a Bachelor’s degree—35.7%. More than 33.2% of the respondents had completed the specialisation course, and 33.8%, apart from completing the specialisation course, had participated in other forms of postgraduate education. Average working experience as a nurse was 18.3 years (SD = 11.6). In the study group, 76.5% were employed under an employment contract in county hospitals (32.1). Detailed sociodemographic data are presented in Table 1.

According to the respondents, the most frequently rationed activities were: providing emotional or psychological support to a patient or the family (1.39; SD = 1.01), the possibility of having an important conversation regarding a particular patient care with another member of an interdisciplinary team or that the conversation was delayed (1.39; SD = 0.99) and the possibility of having an important conversation regarding a particular patient care with an external unit or that the conversation was delayed (1.36; SD = 1.00). The least frequently rationed activities were: administering enteral or parenteral nutrition as prescribed and in accordance with safe practices (0.51; SD = 0.78), administering medication (including intravenous therapy) as prescribed and in accordance with the principles of safe pharmacotherapy (0.52; SD = 0.78) and changing the venous access site, the tube and/or dressings within the prescribed time according to doctor’s orders/standards of the health care facility (0.59; SD = 0.81) (Supplementary Material Table S1).

The mean score of PIRNCA questionnaire was 0.99 (SD = 0.65), which indicates that the respondents ration nursing care rather “rarely”. Average assessment of the quality of patient care amounted to 7.28 in a 0–10 scale (SD = 1.91) and ranged from 0 to 10 points. Average job satisfaction was 6.62 in a 0–10 scale (SD = 2.34) and ranged from 1 to 10 points. On the basis of the analysis of PIRNCA questionnaire, it was found that the study group was characterised by a high appraisal of the quality of nursing care and average level of job satisfaction. The mean score of PSS-10 questionnaire was 23.6 (SD = 5.3). A total of 81.4% of the respondents showed high level of perceived stress, 15.2% an average level and 3.4% a low one (Table 2).

The correlation analysis demonstrated a significant positive correlation (rs = 0.281; *p* < 0.001) between the level of perceived stress (PSS-10) and the level of rationing of nursing care (PIRNCA), which, within the study group, indicates that the higher the level of stress, the higher the level of rationing care. The level of perceived stress (PSS-10) showed significant negative correlation between the level of nursing care (rs = −0.228; *p* < 0.001) and job satisfaction (rs = −0.233; *p* < 0.001), which indicates that the higher the level of stress, the lower the level of nursing care and job satisfaction (Table 3).

On the basis of the analysis, it was found that nurses experiencing a high level of stress were more likely to ration nursing care (respectively: 1.05 vs. 0.77 vs. 0.51; *p* < 0.001), their assessment of nursing care was lower (respectively: 7.20 vs. 7.80 vs. 7.78; *p* < 0.001) and showed lower level of job satisfaction in comparison with nurses with an average or low level of perceived stress (respectively: 6.56 vs. 7.23 vs. 7.50; *p* < 0.001) (Table 4).

The assessment of the relationship between the following variables was conducted: age, marital status, sex, having children, place of residence, education, postgraduate education, work experience, form of employment, place of work and the perceived stress (PSS−10).

Univariate linear regression model analysis showed the effect of marital status, sex, form of employment, place of work and the level of professional burnout on the level of perceived stress as measured with PSS−10 questionnaire. Being in an informal relationship (B = −1.44), employment under civil contract (B = −1.35) and place of work other than in primary health care facility or a hospital (B = −7.67) significantly reduced the level of stress in comparison with being single and employed in other hospitals and primary health care facilities under employment contract. The following were found to markedly increase the level of perceived stress: being male (B = 0.66), working in a regional hospital (B = 3.44) and the level of rationing nursing care (B = 2.30). Higher levels of nursing care quality (B = −0.64) and job satisfaction (B = −0.53) were found to decrease the level of perceived stress (Table 5).

The analysis of the univariate linear regression model showed a marked effect of marital status, place of residence, education, place of work and the level of perceived stress on rationing of the nursing care as assessed with PIRNCA questionnaire. Being in an informal relationship (B = 0.07), living in a city of up to 10,000 inhabitants (B = 0.29), employment in a regional hospital (B = 0.24) and the level of perceives stress (B = 0.03) significantly increased the level of rationing of nursing care in comparison to variables such as being single, living in a countryside, employment in other hospitals or primary health care facilities. Living in a city of up to 100,000 inhabitants (B = −0.16) and holding a PhD title (B = −0.26) showed a significant decrease in the level of rationing of nursing care as compared with living in a large city and having a Bachelor’s degree (Table 6).

## 4. Discussion

Rationing of nursing care is essential in terms of the quality of patient care [33]. It is considered one of the reasons for adverse events and broadly understood harm to a patient; it is also associated with lower job satisfaction [34,35]. Mandal et al. demonstrated that rationing of nursing care is widespread and deeply-rooted in the work environment, presents a risk to occupational health and philosophical foundations of the nursing profession and has a major impact on patient safety [36].

### 4.1. Rationing of Nursing Care

However, even though the most recent studies show that rationing of nursing care is a common problem in the health care system, our own studies demonstrate that nurses rarely ration care.

Similar results are presented in a study by Witczak et al., showing that the average level of missing nursing care amounted to 1.16 (SD = 0.7), and the predictors of rationing of nursing care were connected with the quality of patient care and overall job satisfaction—the factors to be continuously monitored since they indicate the level of rationing of nursing care [37]. The studies by Schubert et al. [38] conducted on surgical, gynaecological and non-invasive treatment wards and by Młynarska et al. [39] showed that nursing care is rarely rationed. Other international studies on nurses from Croatia, the Czech Republic, Slovakia and Poland conducted by Zeleniková et al. showed the average level of rationing of nursing care as within 1.13 and 1.92. This indicates that rationing occurs between “rarely” and “never” [40]. The literature review by Andersson et al. identifies lack of nursing care also in community health care due to organisation and atmosphere of such work [41]. The studies conducted in nursing homes in Switzerland showed a general increase in rationing of nursing care over a study period of 5 years. A marked increase in rationing of the activities of daily living was observed-coefficient 0.47 in 2013 and 0.63 in 2018. The authors believe that the rationing of nursing care is disturbing, especially considering the potential negative effect both on the inpatients as well as the personnel. In the opinion of the authors, regular monitoring of rationing of nursing care should be considered [42]. Willis and Brady conducted a literature review with respect to the effects of rationing of nursing care in Europe, the USA and Oceania in the period 2010–2020. The main effects of lack of care in facilities providing care to adult patients were increased mortality, adverse events and negligence. The same study also identifies a series of causative factors related to working environment in a given ward and low skill level on the part of the personnel [43]. 

### 4.2. Relationship between Rationing of Nursing Care and Sociodemographic Factors

On the basis of our own studies, a relationship between rationing of nursing care and marital status, place of residence, education and place of work was identified.

The results of the effect of sociodemographic variables on rationing of nursing care are not consistent. The studies by Schubert et al. [38] and Papastravou et al. [44] did not show a relationship between sociodemographic variables, i.e., age, education, place of work, duration of employment and rationing of nursing care. Similarly, according to Młynarska et al., sex, age place of residence, education and professional experience were not found to affect rationing of nursing care [38]. Additionally, the study by Jankowska-Polańska et al. did not identify the correlation between education, age, number of places of work, job satisfaction and rationing of care. However, the study showed that fatigue experienced by nurses has an effect on rationing of care [45]. The study conducted by Baszkiewicz et al. shows a positive correlation between the age of the respondent nurses and the level of rationing, i.e., the older the nurse, the more frequent the rationing of nursing care. The same was found with respect to work experience: the longer the seniority, the more frequent the rationing of nursing care [46]. Another multicenter study by Jaworski et al. states that nursing care is most frequently rationed by older nurses as well as those with the lowest work experience [47]. Similarly, Khamisa et al. point to the effect of young age of nurses who, when entering the job market, are inexperienced and ration nursing care more frequently [48]. The aforementioned results do not confirm the findings of the present study.

With respect to nurses in Slovakia, the relationship between age, work experience and rationing of nursing care was not found. However, the correlation between duration of employment in the current position and rationing of care was identified—nurses with working experience in their current position of less than 5 years showed a lower level of rationing of nursing care [49].

Similarly, the present study also identified the effect of the place of work on rationing of care.

### 4.3. Relationship between Rationing of Nursing Care and Job Satisfaction

Since one-third of life is spent at work, job satisfaction is an essential element of everyday life. Satisfaction with work affects self-esteem, which, in turn, is one of the main elements determining nurses’ attitude towards patients. Job satisfaction is the result of a positive appraisal of work, despite inherent negative elements [50]. There are numerous factors affecting job satisfaction: workplace atmosphere, lack of excessive workload, adequate workplace equipment, clear organisation of work, positive relationships within the therapeutic team and with superiors, improved accessibility to professional skills development and higher salary [51]. Uchmanowicz et al. found that job satisfaction and, consequently, rationing of nursing care is also determined by psychological factors such as satisfaction with life and positive life orientation [52].

Our own studies show high appraisals of both the quality of patient care as well as job satisfaction with a low level of average rationing of nursing care.

Similarly, the study by Uchmanowicz et al. indicates that the higher the job satisfaction levels, the lower the level of rationing of nursing care [53].

The analysis of 95,000 nurses demonstrated that patient satisfaction levels were lower in facilities with dissatisfied and burned-out nurses [54]. Other researchers also confirm the aforementioned findings on the negative relationship between rationing of nursing care and job satisfaction [30,55,56,57,58,59]. This implies that lower job satisfaction among nurses coincides with more frequent rationing of care. Undoubtedly, the effect of job satisfaction and the quality of care ought to be continuously monitored to enable implementation of measures aimed at minimising the risk of rationing of nursing care.

### 4.4. Relationship between Rationing of Nursing Care and Stress

Stress affects emotional tension, way of thinking as well as physical health. Continuous stress experienced by nurses may result in their inability to perform their roles, functions and duties, and therefore has a negative effect on the standards of professional nursing care [59,60,61].

According to own studies, with the increase in the level of experienced stress, there is an increased risk of rationing of nursing care, whereas lower level of stress correlates with higher assessment of the quality of care and job satisfaction.

The study conducted by Poghosyana et al. shows that nurses experiencing professional burnout syndrome due to emotional stress may be less likely to provide high quality of care. Continuous close interpersonal contact with patients may predispose nurses to suffer from the burnout syndrome as well as affect rationing of nursing care [62].

Other studies demonstrated that excessive workload is the most common stressor. Furthermore, the said stressor may likely be the reason for low job satisfaction [63,64]. Excessive workload negatively correlates with the assessment of the quality of care [13,65]. Numerous studies confirm the effect of workload on the level of perceived stress [66,67], which, consequently, may predispose nurses to ration care.

Additionally, the present study demonstrated the relationship between stress and marital status, sex, form of employment and place of work.

Similar results were obtained by Alenezi et al., who identified the relationship between stress and marital status and job position and also between stress and nationality and age [68]. Other studies also confirmed the relationship between stress and age. Younger and middle-aged nurses experienced work-related stress more frequently than nurses with many years of experience and increased knowledge of the work environment and professional competence in providing nursing care [69]. Likewise, age was found to be a predictor of stress among nurses working in hospitals and primary health care facilities in Saudi Arabia [59] and Dubai [70], which was not confirmed by our own studies.

### 4.5. Strengths and Limitations

The main strength of the study is the number of respondents; the authors made every effort to ensure random sampling and cross-sectional observational design of the study. The present study also supports the need for a comprehensive approach to rationing of nursing care and stress experienced by nurses as well as determining factors.

However, there are some limitations to the study. It was conducted in three selected provinces of one country; therefore, it is difficult to draw conclusions regarding the general population of nurses in Poland or worldwide. In order to confirm the results of the present study, the studies are to be continued not only in other provinces in Poland but also on an international scale. 

## 5. Conclusions

1. Stress in nurses has a negative effect on rationing of nursing care and job satisfaction. It is recommended that actions aimed at developing effective stress coping skills be implemented as early as at the stage of training to become a nurse.
2. Factors such as marital status, sex, form of employment, place of employment and the level of professional burnout syndrome may have an influence on the level of experienced stress. In turn, the level of experienced stress, marital status, education, place of work as well as the place of residence may have an effect on rationing of nursing care and, consequently, affect the quality of care.

## Figures and Tables

**Table 1:** Sociodemographic and clinical characteristics of the study group.

Variable		x̅	Me	Min	Max	Q1	Q3	SD
**Age (ears) (*n* = 567)**		42.2	45.0	22.0	67.0	33.0	50.0	10.8
**Work experience (years) (*n* = 567)**		18.3	20.0	0.0	46.0	6.0	28.0	11.6
Variable	Variable category	*n*			%			
Sex (*n* = 567)	Female	427			75.3			
	Male	140			24.7			
Marital status (*n* = 278)	Informal relationship	47			16.9			
	Formal relationship	171			61.5			
	Single	60			21.6			
Do you have children? (*n* = 150)	No children	76			50.7			
	Children up to 18 years of age	29			19.3			
	Children over 18 years of age	45			30.0			
Place or residence (*n* = 278)	Countryside	47			16.9			
	City of up to 10,000 inhabitants	45			16.2			
	City of up to 100,000 inhabitants	89			32.0			
	City of more than 100,000 inhabitants	97			34.9			
Education (*n* = 356)	Nurse	98			27.5			
	Bachelor’s degree	127			35.7			
	Master’s degree	97			27.2			
	PhD	34			9.6			
Postgraduate education (*n* = 229)	Not applicable	43			18.8			
	Courses	36			15.7			
	Specialisation	76			33.2			
	Specialisation, courses	74			32.3			
Form of employment (*n* = 149)	Civil contract	35			23.5			
	Employment contract	114			76.5			
Facility of employment (*n* = 340)	Primary health care facility	70			20.6			
	County hospital	109			32.1			
	Teaching hospital	90			26.4			
	Regional hospital	67			19.7			
	Other	4			1.2			

x̅—mean; Me—median; Q1—first quartile; Q3—third quartile; Min—minimum value; Max—maximum value; SD—standard deviation; *n*—number of individuals; %—percent of individuals.

**Table 2:** PIRNCA questionnaire results and level of perceived stress among nurses participating in the study.

Variable		x̅	Me	Min	Max	Q1	Q3	SD
**PIRNCA (*n* = 557)**		0.99	0.94	0.00	3.00	0.50	1.39	0.65
**Assessment of standard quality of nursing care in your ward? (*n* = 564)**		7.28	8.00	1.00	10.0	6.00	9.00	1.91
**Assessment of the level of satisfaction with the current nursing job, considering all aspects of the job, including your values, ideals and goals? (*n* = 564)**		6.62	7.00	0.00	10.0	5.00	8.50	2.34
**PSS-10 (*n* = 528)**		23.6	23.0	8.0	40.0	20.0	26.0	5.3
**Variable**	Variable category	*n*			%			
**PSS-10 (*n* = 528)**	Low	18			3.4			
	Medium	80			15.2			
	High	430			81.4			

x̅—mean; Me—median; Q1—first quartile; Q3—third quartile; Min—minimum value; Max—maximum value; SD—standard deviation; *n*—number of individuals; %—percent of individuals.

**Table 3:** Results of the correlation analysis of the perceived stress among the nurses participating in the study and rationing of nursing care.

Variable	PSS-10	
	r_s_	*p*
**PIRNCA**	0.281	<0.001
**Assessment of standard quality of nursing care in your ward?**	−0.228	<0.001
**Assessment of the level of satisfaction with the current nursing job, considering all aspects of the job, including your values, ideals and goals?**	−0.233	<0.001

r_s_—Spearman correlation coefficient.

**Table 4:** Results of the correlation analysis of PSS-10 and PIRNCA in groups showing differences in terms of the stress levels.

Variable		PIRNCA							Value *p* *
		x̅	Me	Min	Max	Q1	Q3	SD	
**PSS-10**	Low	0.51	0.44	0.00	1.69	0.20	0.74	0.44	<0.001
	Medium	0.77	0.68	0.00	2.65	0.28	1.02	0.63	
	High	1.05	1.00	0.00	3.00	0.59	1.45	0.65	
		**Assessment of standard quality of nursing care in your ward?**							
**PSS-10**	Low	7.78	8.00	4.00	10.00	7.00	9.00	1.63	0.049
	Medium	7.80	8.00	4.00	10.00	7.00	9.00	1.56	
	High	7.20	8.00	1.00	10.00	6.00	9.00	1.95	
		**Assessment of the level of satisfaction with the current nursing job, considering all aspects of the job, including your values, ideals and goals?**							
**PSS-10**	Low	7.50	8.00	2.00	10.00	7.00	9.00	2.20	0.026
	Medium	7.23	7.50	3.00	10.00	5.00	9.00	2.18	
	High	6.56	7.00	0.00	10.00	5.00	8.00	2.35	

x̅—mean; Me—median; Q1—first quartile; Q3—third quartile; Min—minimum value; Max—maximum value; SD—standard deviation; * Kruskal-Wallis ANOVA test.

**Table 5:** Results of linear regression analysis of the effect of the selected variables on the perceived level of stress (PSS−10).

	PSS−10 Total Score					
		B	SE	t	*p*-Value	ß
**Age**		0.00	0.02	−0.06	0.951	0.00
**Marital status (ref. formal relationship)**	Informal relationship	−1.44	0.64	−2.24	0.026	−0.19
	Single	1.01	0.60	1.69	0.092	0.14
**Sex (ref. Female)**	Male	0.66	0.26	2.50	0.013	0.11
**Do you have children?** **(ref. no children)**	Up to 18 years of age	−0.52	0.87	−0.59	0.554	−0.06
	Over 18 years of age	0.79	0.78	1.02	0.309	0.11
**Place of residence (ref. city of more than 100,000 inhabitants)**	Countryside	0.19	0.71	0.26	0.794	0.02
	City up to 10,000 inhabitants	1.21	0.72	1.68	0.095	0.14
	City up to 100,000 inhabitants	0.20	0.58	0.35	0.727	0.03
**Education (ref. Bachelor’s degree)**	Nurse	0.76	0.60	1.25	0.211	0.10
	Master’s degree	0.02	0.61	0.02	0.980	0.00
	PhD	−0.63	0.95	−0.67	0.506	−0.06
**Postgraduate education (ref. specialisation, courses)**	Not applicable	−0.94	0.82	−1.14	0.254	−0.10
	Courses	1.14	0.87	1.30	0.195	0.11
	Specialisation	0.34	0.69	0.49	0.621	0.04
**Work experience**		−0.01	0.02	−0.26	0.797	−0.01
**Form of employment (ref. employment contract)**	Civil contract	−1.35	0.60	−2.25	0.026	−0.18
**Facility of employment (ref. county hospital)**	Primary health care facility	0.65	0.83	0.78	0.435	0.08
	Teaching hospital	0.90	0.89	1.01	0.313	0.11
	Regional hospital	3.44	0.84	4.09	0.000	0.44
	Other	−7.67	2.32	−3.30	0.001	−0.65
**PIRNCA - total score**		2.30	0.34	6.67	0.000	0.28
**Assessment of standard quality of nursing care in your ward?**		−0.64	0.12	−5.36	0.000	−0.23
**Assessment of the level of satisfaction with the current nursing job, considering all aspects of the job, including your values, ideals and goals?**		−0.53	0.10	−5.49	0.000	−0.23

B—unstandardized regression coefficient B; SE—standard error; t—B/standard error; ß—standardized regression coefficient ß.

**Table 6:** Results of linear regression analysis of the effect of the selected variables on the level of nursing care rationing (PIRNCA).

	Kolumna2	PIRNCA—Total Score				
		B	SE	T	*p*-value	ß
**Age**		0.00	0.00	−0.71	0.477	−0.03
**Marital status (ref. formal relationship)**	Informal relationship	0.07	0.03	2.20	0.028	0.09
	Single	0.00	0.07	−0.06	0.955	0.00
**Sex (ref. Female)**	Male	0.04	0.07	0.63	0.530	0.05
**Do you have children?** **(ref. no children)**	up to 18 years of age	−0.08	0.10	−0.83	0.409	−0.09
	over 18 years of age	0.14	0.09	1.64	0.104	0.18
**Place of residence (ref. city of more than 100,000 inhabitants)**	Countryside	0.03	0.08	0.39	0.696	0.03
	City up to 10,000 inhabitants	0.29	0.08	3.51	0.001	0.29
	City up to 100,000 inhabitants	−0.16	0.07	−2.48	0.014	−0.20
**Education (ref. Bachelor’s degree)**	Nurse	0.04	0.06	0.70	0.484	0.05
	Master’s degree	0.10	0.06	1.55	0.123	0.12
	PhD	−0.26	0.09	−2.72	0.007	−0.23
**Postgraduate education (ref. specialisation, courses)**	Not applicable	0.06	0.09	0.67	0.506	0.06
	Courses	−0.07	0.10	−0.78	0.437	−0.07
	Specialisation	0.08	0.08	1.09	0.277	0.09
**Work experience**		0.00	0.00	−0.98	0.328	−0.04
**Form of employment (ref. employment contract)**	Civil contract	0.00	0.07	0.00	0.998	0.00
**Facility of employment (ref. county hospital)**	Primary health care facility	−0.07	0.10	−0.78	0.438	−0.08
	Teaching hospital	0.02	0.09	0.21	0.834	0.02
	County hospital	0.24	0.10	2.50	0.013	0.25
	Other	−0.40	0.27	−1.51	0.133	−0.29
**PSS−10—total**		0.03	0.01	6.67	0.000	0.28

B—unstandardized regression coefficient B; SE—standard error; T—B/standard error; ß—standardized regression coefficient ß.

## Data Availability

The datasets generated during and/or analysed during the current study may be made available by the corresponding author on request.

## Supplementary Materials

The following supporting information can be downloaded at: https://www.mdpi.com/article/10.3390/ijerph20032414/s1, Table S1: PIRNCA questionnaire- detailed results.
