# Supplementary material for: Assessment of The Effect of Stress, Sociodemographic Variables and Work-Related Factors on Rationing of Nursing Care
Source: Int J Environ Res Public Health. 2023 Jan 29;20(3):2414. doi: 10.3390/ijerph20032414 (PMC9915080; doi:10.3390/ijerph20032414)
Supplement: Supplementary file 1 [file ijerph-20-02414-s001.zip › ijerph-2091005-supplementary.pdf]

Supplementary Materials

Table S1. PIRNCA questionnaire- detailed results

How often did it happen in the last seven working days?	$\bar{x}$	Me	Min	Max	Q1	Q3	SD
You could not carry out routine hygiene care of patients (e.g. bath, oral hygiene, dental care) or ensure that the task was performed by delegating it to another staff member	1.05	1.00	0.00	3.00	0.00	2.00	0.94
You could not carry out routine skin care in patients or ensure that the task was performed by delegating it to another staff member?	1.03	1.00	0.00	3.00	0.00	2.00	0.92
You could not change the bedding stained with blood or body fluids in a timely manner or ensure that the task was performed by delegating it to another staff member?	1.11	1.00	0.00	3.00	0.00	2.00	0.96
You could not assist a patient in need of walking or ensure that the task was performed by delegating it to another staff member?	1.21	1.00	0.00	3.00	0.00	2.00	1.00
You could not mobilise a patient or change the position of a patient with limited mobility or ensure that the task was performed by delegating it to another staff member?	1.23	1.00	0.00	3.00	0.00	2.00	0.98
You could not provide quick assistance to a patient with emptying the bowel or bladder (e.g. using a bedpan, toilet chair or in the bathroom) or ensure that the task was performed by delegating it to another staff member?	1.06	1.00	0.00	3.00	0.00	2.00	0.93
You could not provide adequate assistance to a patient unable to eat or drink unassisted, regardless of the manner of food intake or ensure that the task was performed by delegating it to another staff member?	0.98	1.00	0.00	3.00	0.00	2.00	0.93
You could not implement measures promoting physical comfort (e.g. timely administration of painkillers, adjusting temperature, back or neck massage) or ensure that the task was performed by delegating it to another staff member?	1.02	1.00	0.00	3.00	0.00	2.00	0.95
You could not administer medication (including intravenous therapy) as prescribed and in accordance with the principles of safe pharmacotherapy?	0.52	0.00	0.00	3.00	0.00	1.00	0.78
You could not administer enteral or parenteral nutrition as prescribed and in accordance with safe practices?	0.51	0.00	0.00	3.00	0.00	1.00	0.78
You could not provide care of wounds (including changing dressing) according to doctor's orders/ standards of the healthcare facility or when you find it necessary?	0.60	0.00	0.00	3.00	0.00	1.00	0.83
You could not change the venous access site, the tube and/or dressings within prescribed time according to	0.59	0.00	0.00	3.00	0.00	1.00	0.81

doctor's orders/ standards of the healthcare facility or when you find it necessary?							
You could not fully adhere to the guidelines for safe handling of the patient (e.g. the use of assistive equipment and lifts and/or additional personnel)?	0.95	1.00	0.00	3.00	0.00	2.00	1.00
You could not fully adhere to the guidelines for infection control (e.g. hand hygiene, aseptic technique, isolation)?	0.66	0.00	0.00	3.00	0.00	1.00	0.87
You could not provide enough time, which in your opinion was needed, for educating the patient and/or the family?	1.18	1.00	0.00	3.00	0.00	2.00	1.05
You could not prepare patients properly for treatment, tests or procedures?	0.80	1.00	0.00	3.00	0.00	1.00	0.89
You could not provide adequate level of emotional or psychological support to a patient or the family as needed?	1.39	1.00	0.00	3.00	1.00	2.00	1.01
You could not monitor a patient's physiological condition (e.g. vital signs, laboratory values) according to doctor's orders/ standards of the healthcare facility or when you find it necessary?	0.83	1.00	0.00	3.00	0.00	1.00	0.92
You could not monitor the emotions and behaviour of a patient (e.g. medication compliance, eating habits, social interactions or mood) according to doctor's orders/ standards of the healthcare facility or when you find it necessary?	1.16	1.00	0.00	3.00	0.00	2.00	0.99
You could not monitor the physical safety of a patient according to doctor's orders/ standards of the healthcare facility or when you find it necessary?	1.02	1.00	0.00	3.00	0.00	2.00	0.93
You could not monitor changes in the condition of a patient, there were missed requests for intervention regarding a patient (including assessment or referral) or unclear orders?	0.99	1.00	0.00	3.00	0.00	2.00	0.89
You had to keep a patient or family member waiting for more than 5 minutes from the moment they signalled the request (e.g. by alert light)?	1.35	1.00	0.00	3.00	1.00	2.00	1.00
You could not have an important conversation with another member of the interdisciplinary team regarding patient care or the conversation was delayed?	1.39	1.00	0.00	3.00	1.00	2.00	0.99
You could not have an important conversation with an external unit regarding patient care or the conversation was delayed?	1.36	1.00	0.00	3.00	1.00	2.00	1.00
You could not have an important conversation with a patient of family regarding their needs or instructions related to hospital discharge, or the conversation was delayed?	1.30	1.00	0.00	3.00	1.00	2.00	0.98
You could not provide adequate monitoring or tracking the performance of tasks delegated to another staff member?	1.24	1.00	0.00	3.00	0.00	2.00	0.97

You could not review the interdisciplinary records of a patient to get comprehensive patient data?	1.24	1.00	0.00	3.00	0.00	2.00	0.97
You could not document the initial or modified care plan?	1.06	1.00	0.00	3.00	0.00	2.00	0.94
You could not document all activities regarding the assessment and monitoring of a patient's condition?	0.95	1.00	0.00	3.00	0.00	2.00	0.91
You could not document in detail the entire process of providing nursing care?	0.96	1.00	0.00	3.00	0.00	2.00	0.93
You could not properly assess the nursing care plan (using critical thinking) to determine the validity and/or effectiveness of an intervention and to introduce recommended changes?	1.04	1.00	0.00	3.00	0.00	2.00	0.92
